# Action-Shapers and Their Neuro-Immunological Foundations

**DOI:** 10.3389/fpsyg.2022.917876

**Published:** 2022-07-15

**Authors:** Otto Paans, Boukje Ehlen

**Affiliations:** Independent Researcher, Simpelveld, Netherlands

**Keywords:** neuro-immunology, philosophy, thought-shaping, action-shaping, intentional abilities

## Abstract

Not all our intentions translate into actions, as our capacity to act may be influenced by a variety of mental and biochemical factors. In this article, we present a comprehensive account of how neuro-immunological processes affect our intentional abilities and our capacity to act. We do so by extending the theory of thought-shapers (TTS) through the notion of action-shapers and combining this theory with the essential embodiment thesis (EE). This thesis about the mind-body relation says that human minds are necessarily and completely embodied. Action-shapers dynamically constitute the action-space of individuals, affecting their capacity to take action or to select one course of action over another. We highlight the effects and interactions of neuro-immunological effective processes in the body to demonstrate how they shape the action-space. In this article, we consider neuro-immunological effective processes that influence the gut-brain axis, chronic stress, high levels of sugar intake, the amygdala and the effects of prolonged stress. We investigate the effects of these processes on the perception and on the capacity to form intentions and act on them. We conclude the paper by providing a concise account of action-shapers, in which we attempt to summarize the line of argumentation and provide suggestions for further research.

## Introduction

Not all of our thoughts or intentions are translated into actions. We may play around with the thought of directing this or that snide remark at our boss, or of finally taking up jogging to lose some weight, but many of those intentions are not converted into actions. But why is this so? Why do some resolutions only seldomly translate into actions, even while we want to take action at a conscious or self-conscious level? At the very least, we often play around with thoughts, while we never work up the effort to put them into action. A person might *really* want to take up jogging, but not arrive at the point where he puts on running shoes.

This article deals with cases in which a person has sufficient reason to perform action A and at some level desires to perform action A, but somehow the capacity to convert this desire into an actual performance becomes impaired, so action A is not performed. However, such cases may occur due to various factors. In the cases we consider here, the capacity to take action is impaired by factors that are related to our embodiment.

The hypothesis that we develop in this article is that this phenomenon occurs due to the effect of action-shapers. Furthermore, we argue that action-shapers are the natural counterparts of thought-shapers (Hanna and Paans, 2021). Thought-shapers are mental representations that specifically shape our essentially embodied human thinking processes. Action-shapers habituate and impact our patterns of performing actions. Especially, we consider the formation of action-shapers from a neuro-immunological point of view. Given the emphasis on neurological processes in the analytic philosophy of mind (Smith Churchland, 1989; Goldblum, 2004; Churchland, 2013; Wolfe, 2014), it is not surprising that the role of other biological systems that operate in our bodies are somewhat undertheorized. To be sure, this orientation derives largely form the influence of mechanistic models to explain biological processes and the functioning of nervous systems alike (Ochs, 2004; Hanna and Paans, 2020; Pecere, 2020). However, we side here with those that question attempts to ascribe too much weight to neurology alone (Roskies, 2007). To remedy this situation, we would like to highlight the links between what we call *neuro-immunological effective processes* and their effects on our capacity to “take action.”

A given person can want to perform A, but nevertheless not be able to effectuate this intention. In other words, willing is not always enough for performing A. As there are intimate links between the effects of thought-shapers and action-shapers, the account we provide here showcases some of the complexity involved and provides the first outline of what might be a refined understanding of our capacity for acting.

The discussion is structured as follows: in section “Definitions,” we introduce three definitions to demarcate the scope of our argument. This is followed by a concise consideration about a model of human intentional action derived from Aristotle’s *Physics* (Reeve, 2018, p. 149), namely what we may characterize as The Domino Model (TDM), the implications of which are concisely described in Chisholm (2013). The next section provides a preliminary definition of action-shapers. Section “Action-Shapers: A Preliminary Sketch” introduces some background on neuro-immunological effective processes and highlights with a few examples how these processes function as action-shapers. In section “Neuro-Immunological Effective Processes,” we discus some implications of our view, and summarize the argument.

It should be clear that—given the recency of the theory of thought-shapers– we cannot provide an exhaustive account of thought-shaping and action-shaping. Instead, we sketch the main contours of a new line of inquiry that diverges importantly from reductive materialism or physicalism in the philosophy of mind. In particular, we aim here to provide a synoptic rather than analytic account. That is, we attempt to align various types of evidence in order to “see them together” (Gare, 2014, p. 320), so as to provide a theory of action-shaping.

## Definitions

*The essential embodiment thesis* (EE) about the mind-body relation says that human minds are necessarily and completely embodied, and identical to the complex dynamic, spontaneously activating, intentional-action-guiding, global structures of suitably complex living organisms belonging to the human species, i.e., human animals (Hanna and Maiese, 2009). With regard to the hypothesis we develop, EE is important because we cannot neatly distinguish between our bodily state and occurrent biochemical processes and the formation of beliefs and/or desires. Whereas the Western philosophical distinction has often retained Cartesian dualism in some form or the other, EE rejects this distinction, instead implying that whenever we think about the mind, we cannot do so without the body, or even that this categorization is not correct.

*Shaping* is the term we use for processes that partially (but not wholly) causally determine, dynamically form, and normatively guide our essentially embodied human minds and lives (Maiese and Hanna, 2019).

*Thought-shapers* are mental representations, especially including analogies, images, schemata, stereotypes, symbols, and templates, that specifically shape our essentially embodied human thinking processes; and *the theory of thought-shapers* (TTS) about human thinking says that our essentially embodied human thinking processes are either, (a) shaped negatively by mechanical, constrictive thought-shapers, or (b) shaped positively by organic, generative thought-shapers (Hanna and Paans, 2021).

Thought-shapers operate pre-consciously and schematically combine essentially non-conceptual and conceptual contents. This feature makes it hard to catch them “at work,” as they shape conscious thought and disposition continuously, while we are often only intermittently or not at all aware of them. Given the fact that thought-shapers are mental representations with topological and processual properties, we access them primarily through our conative and cognitive attitudes of imagination.

The cognitive attitude is successful when the contents of the mind are reflected in the world, that is, when there is a match between what is mentally represented and what is perceptually present. Conversely, the conative attitude is successful when the contents of the world are satisfied in the mind (Kind, 2017a, p. 5; Kind, 2017b). It follows that *believing* is a paradigmatic cognitive attitude, while *desiring* is a paradigmatic conative attitude. In the first case, the belief is justified when it matches a state of affairs in the manifestly real world. In the second case, the desire is feasible when it is directed toward some possible state of affairs that could obtain at some point in time. So, thought-shapers straddle the mind-world connection in both directions, and consequently, influence and orient the domains of belief, desire and intention through imaginative force.

## Human Intentional Action: The Domino Model

Let’s consider a picture of human intentional action that is—in its many variations—quite widespread since at least the time of Aristotle. We refer to it as “The Domino Model” (TDM). According to TDM, an action is preceded by a decision to take action, which is in turn preceded by a motive or reason for deciding to act (Chisholm, 2013; Reeve, 2018, p. 149). So, TDM takes the form of a causal explanatory chain. When someone is said to desire to raise his arm, we have to explain not just the motives for intending to do so, but also where the motive came from. Answering this question by referring to some primal will or volition does little to shed light on the matter, as the question then becomes what the cause of the primal will actually is.

To complicate things further, some desires seem to arise spontaneously, and no motive or reason can be given for them. And, equally, some intentions cannot be reduced to motives on a one-one basis.

Consider the following scenario. Green is just about to raise his arm at time T_1_, in order to perform an action—for instance, wave to someone else, reach for the remote control, stop a child from running out into the street, or to simply stretch his arm. If Green does indeed raise his arm to perform that particular action, then his arm-raising is his *basic* intentional act.

In this context, for whatever reason, Green *spontaneously effectively desires* to raise his arm. If the desire is *effective*, then it is causally sufficient to move him all the way to action. Assuming that other things remain equal in this context, Green *will* raise his arm by way of satisfying that effective desire. If Green *hadn’t effectively desired to raise his arm* in that context, then Green’s *arm would not have been raised* in that context.

So, Green, and no one else, is *the ultimate source* of his arm-raising, basic intentional act at T_1_: he could *either* have raised his arm or have *not* raised his arm, depending on the repertoire of spontaneous effective desires at T_1_ and the moments directly preceding it. No deterministic or indeterministic mechanical system caused Green to desire, choose, or act, and therefore Mr. Green possesses what is called *source-incompatibilist free will* (Hanna, 2018).

In an alternative scenario devised by (Frankfurt, 1995b) an evil entity (called Black) manipulates Green in such a way that he will raise his arm at T_1_, completely in line with Black’s intentions. Yet, Green does not know this, and raises his arm nevertheless, without Black actually manipulating him.

In this context, Green formally *lacks* the ability to choose or do otherwise due to Black’s manipulation. But provided that he *already* spontaneously and independently effectively desired to raise his arm, Green is *still* the ultimate source of his freely willed arm-raising intentional act, despite his having no ability to choose or do “otherwise” (Frankfurt, 1995a,b).

Frankfurt’s thought experiment was designed to show that human intentional agents can be the ultimate sources of their freely willed basic intentional acts in a given context, and be causally and morally responsible for those acts, even if (I) they actually lack the ability to choose or do otherwise in that context *and* provided that (II) they’re actually capable of spontaneous effective desiring in that context.

As already said, TDM takes the form of a backwards-stretching causal explanatory chain: Mr. Green is said to be desiring to raise his arm at T_1_ and consequently, the discussion unfolds about what the constraints on his performing this action are. What TDM does not explain is *how* the desires came about in the first place. Desires may indeed arise spontaneously, due to someone’s predisposition, preference etc. But even if we recognize this as a fact, we require a theory that explains this emergence in more detail and relates it to the conditions occurring in our physical embodiment. As indicated, if we opt for explanatory models that only deal with motives, desires and some form of primal volition, we keep the fact and influence of our embodiment and evolutionary history out of the explanation, and slide back in a modern-day version of Cartesian dualism—one in which motives and desires are not linked to biological, immunological and biochemical processes at all. With these fundamentals in place, we can establish a few points of departure for our discussion:

a) In some contexts, some spontaneously willed desires arise outside the view of our self-conscious minds (i.e., they arise without any self-conscious deliberation or any realization that they are forming).
b) Human agents possess free will in some contexts, although, as per (a) there must be an additional factor or set of factors that shapes the emergence of (I) desires, whether spontaneous or not, and also shapes (II) the effectiveness of those desires to turn into actions.

As per (a) and (b) self-conscious deliberative motives or reasons are not the *only* factors that prompt people to choose or act or abstain from choosing or acting. I can consciously form the intention to take up jogging upon perceiving that I have gained weight, but I may be unable to put this intention into action.

The Frankfurt-style thought experiment does not establish that Black robs Green from choosing to do otherwise, but simply *narrows down* the options that Green has at his disposal at T_1_. So, while Green strictly speaking does not lose his ability to choose, his range of options becomes so narrow that for all intents and purposes, it appears that Green has only one option left over to choose from, although strictly speaking he does not lose his *capacity for choice* itself.

So, as per (a–d) TDM overlooks the connection between intention and act: Green can still desire to raise his arm but be unable to effectuate this action. In that case, the intention is effective, but its performance is not.

Concluding, TDM leaves at least two issues unanswered: (A) how it is that some desires arise seemingly spontaneously, apart from self-conscious awareness and rational deliberation, and (B) how it is that even effective desires can be present but are not carried over into actions. Note that the conceptual issues surrounding the so-called “classical theory of action” (Frankfurt, 1995b) are outside the scope of this article, although there are many thematic overlaps that could be identified.

By extending the basic concept behind TTS, we can possibly provide a promising alternative that is suitable to address these issues. We should add a caveat here with regard to the scope of this paper: we discuss here the hypothesis that action-shapers steer and shape the human capacity to translate intentions into actions. Of course, there is an evolutionary process underlying the volitional capacities of human beings, but we leave the manifold influences of that developmental history largely outside our discussion for reasons of space and focus. However, it should be kept in mind that many links can (and should) be drawn between the evolutionary history of our volitional capacities and the hypothesis of action-shapers.

## Action-Shapers: A Preliminary Sketch

According to TTS, thought-shapers shape human thinking in various ways, some of which are mechanical and constrictive, others of which are organic and generative. Due to their shaping activity, thought-shapers partially (but not wholly) causally determine, effectively direct, guide, influence, and orient the emergence of thoughts. If they did not, TTS would have no real-world impact whatsoever (Hanna and Paans, 2021). The constrictive character of mechanist thought-shapers stems from the fact that an idealized model or point of reference is used to specify, interpret and perceptually color various context-sensitive datums, even when there is no justification for doing so. By contrast, generative thought-shapers function in an opposite manner:

[B]y “installing” human thinking in inherently *re-configurable* and *re-patternable* “grooves,” self-consciously unlock, liberate, and sustain creative and productive human thinking. A characteristic feature of generative thought-shapers is that they possess not only effective, true, flexible application to a proper domain of content, but also effective, true, flexible *re-application* or *repurposing*, across several or even unrestrictedly many *different* domains of content, yet *without* being infinitely malleable, ambiguous, or vague (Hanna and Paans, 2021, p. 24).

Thought-shapers pre-self-consciously shape thoughts and therefore they also pre-self-consciously shape intentions, desires, beliefs and volitions. But desires can become effective and turn into actions. So, we can say that thought-shapers at some point develop or extend into their action-oriented counterparts that translate desires into real physical actions.

We call these action-oriented counterparts *action-shapers*. Likewise, we call the context-specific operating environment of an action-shaper *the action-space*. Referring back to the example of Green and Black, even though he’s a human intentional agent with source-incompatibilist free will, Green nevertheless spontaneously effectively desires, chooses, and acts according to some action-shaper within an action-space that *partially (but not wholly) causally* determines, forms, and normatively guides his arm-raising act.

The “partially-but-not-wholly” clause is important. Not all actions are linearly partially caused by action-shapers, just as not all thoughts are linearly partially caused by thought-shapers. Similarly, actions are not completely causally determined by action-shapers, just as thoughts are not completely causally determined by thought-shapers. If we were to hold that view, we would be thrown back into a kind of bleak, deterministic, and mechanistic picture about human agency.

Thought-shapers primarily exercise their influence through our capacity for imagination (Hanna and Paans, 2021). By contrast, action-shapers exert their influence primarily via desires, affects or emotions. This is not to say that thought-shapers and action-shapers function in isolation, as naturally, their effects revert into one another. And just as thought-shapers might be closely bound up with passions or emotions, so action-shapers might well involve mental imagery. So, the primary modes of exerting influence differ for both thought-shapers and action-shapers but are not mutually exclusive.

A far better way of thinking about both thought- shapers and action-shapers is to regard them as exerting a salient yet non-deterministic influence throughout our representational capacities involved in our self-image and our perception of the world, exercised via the imagination or desires, intentions and affects. In the cases of bad thought-shaping and bad action-shaping, we might imagine them as being like the archetypical malevolent advisor who whispers half-truths and suggestions into a king’s ear. At no point does the advisor literally tell the king what to do, but he definitely directs the course of action that the king will take, without, however, taking away the king’s capacity for free choice. In the cases of good thought-shaping and good action-shaping, we can imagine them as being like the equally archetypal benevolent advisor who offers constructive advice to the king.

In an analogous manner, action-shapers do not remove one’s capacity for freely choosing to perform an action or abstaining from doing so, but instead they act as diffuse yet salient causal influences, motivators, detractors, moderating or aggravating forces on the translation from desire into action.

A person’s action space is the array of possible actions he or she can perform at T_1,_ given the influential presence of various contextual conditions and weak yet salient causal forces, such as thought-shapers or action-shapers, in play at T_1_. If we return to the example of Black and Green, we can imagine that Green finds himself originally in a very broad action space. He can lift his arm if he desires to do so, without any constraint. But if Black starts to tamper with Green’s ability to translate desires into actions, or if Black manipulates Green’s preferences to choose option A over option B in similar circumstances, then we can see that the action space of Green is narrowed down, possibly for the worse. And, if we imagine an extreme case, then we can even imagine that Green’s action space is narrowed down to the degree that he has one and only one choice in that context.

We must add one important qualifier. As defined above, thought-shapers and action-shapers alike influence the orientation of beliefs, desires and intentions. So, while Green might *believe* that he cannot raise his arm, he might be *actually* able to perform that act anyway. But because he does not believe that he can, he abstains from trying. So, while the action-space from Green’s own point of view seems very constrained, objectively it might be much broader than he imagines.

Although there are certainly cases where a narrow action-space can be bad, it is worth noting that the opposite is also true: in some cases, a certain focus or single-mindedness can be intensely rewarding, for instance in engaging in creative work. Alternatively, someone who witnesses a swimmer drowning, and chooses to risk his own life by jumping in the water and saving the swimmer might well say that he felt that he “had no other choice,” although formally he could have walked away. In such cases, the narrowing down of the action-space is a positive feature.

Thought-shapers and action-shapers mutually influence each other. A thought-shaper whose content is expressible as “I will never be good enough to perform action X,” may lead to the formation of a “limiting belief,” (Fannin and Williams, 2012; Lipton, 2015; Wagner et al., 2015) that in turn feeds into a defeatist action-shaper. The action-shaper inflects the action space in such a way that it seems to exclude the possibility of performing X. While without the thought-shaping influence, the action space might well have included the possibility of performing X at least in theory, with the action-shaper imposed on it, the action-space appears as excluding the possibility of actually performing X.

Since a limiting belief is a belief that is reflexively directed toward oneself, it follows that it has a close relation to self-representation and indexicality: does the person to whom this happens *really not know* that X can be performed at T_1_? Yes, he or she does, but does not believe that the possibility is a real one *for him or her right here and right now*. Performing action X exists as a theoretical possibility but appears not as part of one’s repertoire of available actions at T_1_. And consequently, bodily systems do not align in such a way that the action is actually carried out. Alternatively, we may say that limiting beliefs influence one’s *attributional style* with regard to the relationship between self and world (Alloy and Abramson, 1979; Seligman et al., 1979), as has been already established in patients suffering from depression.

According to TTS, the formation of beliefs, including “limiting beliefs” is directly and indeed intimately related to the non-conceptual, self-representing features of both thought- and action-shaping (Hanna and Paans, 2021). One could say that the influence of both thought-shaping and action-shaping actively create and sustain the dynamic image one entertains about oneself on both the non-conceptual and conceptual levels.

The influences between thought-shapers, action-shapers, “limiting beliefs,” and self-presentation can interact in multiple directions. Thought-shapers can narrow or broaden the array of action-shapers. For instance, recent research (Arnaldo et al., 2022) has shown that once our allostatic load, that is, the stress on so-called allostatic setpoints, or stable reference points within the environment (Sterling et al., 1988) is exceeded, this can contribute to the development of disorders related to depression. These findings coincide partially with our concept of action-shapers that positively or negatively affect one’s possibilities for taking action and/or performing acts effectively. So, action-shapers can be seen as dynamically contoured depressants or stimulants on the decision-making involved in performing or abstaining from performing an action.

Indeed, whenever someone’s action-space is exceedingly narrow, that person will effectively desire, choose, and act as if “there’s simply no other option,” even if that is objectively false. Many problems in thinking about free will hinge on this point: if we say, truly, that a person “could have done otherwise” in a given situation, we mean to say that the action-space of that person was in reality broader than the actual choice being made; yet the Frankfurt-cases show that the actual lack of such options is no impediment either to genuine source-incompatibilist free will or to genuine causal or moral responsibility (Frankfurt, 1995b).

So far, we have provided a summary sketch of the relation of thought-shapers and actions-shapers to an individual action space. However, as per EE, human minds are necessarily and completely embodied, and identical to the complex dynamic, spontaneously activating, intentional-action-guiding, global structures of suitably complex living organisms belonging to the human species (Hanna and Maiese, 2009). If we combine TTS and EE, we have to explain, at least, how action-shapers are not just the result of rational deliberation or self-conscious mental action. If we opt for that route, then we run into the fundamental issue that TDM does not address: namely, that some motives, desires and intentions seem to arise spontaneously, and cannot be explained on the grounds of rational deliberation alone. Nor can we fall back on some fundamental force—a primal will or primal volition perhaps—that has to perform all the explanatory work. If we were to opt for that route, then we would have to inquire what causes or shapes the primal will or volition, thereby falling into an infinite regress.

In combining the core commitments of TTS and EE, we end up with the following set of statements: (a) human minds are necessarily and completely embodied, and identical to the complex dynamic, spontaneously activating, intentional-action-guiding, global structures of suitably complex living organisms belonging to the human species and (b) that our essentially embodied human thinking processes are either (I) shaped negatively by mechanical, constrictive thought-shapers, or (II) shaped positively by organic, generative thought-shapers. Note that in some cases, constrictive and generative thought-shapers may be simultaneously active within a single organism. So, one could believe that one would never be able to run a half-marathon (constrictive), while at the same time attempting to take up cycling (generative).

With these fundamentals in place can construct a new and more comprehensive account of human intentional action by combining EE and TTS. That would be an account that is fully organicist/organism-oriented, taking into account how neuro-immunological processes influence our capacity for action-taking. As we stressed before, it is important to establish the connections between the various bodily processes that influence our volitional capacities and the first-person, subjective experience that they create in order to provide an alternative to TDM. Moreover, if we can explain in a summary way how such processes can turn out to be generative or constrictive, TTS is supported with a theoretical foundation that can be used for formulating hypotheses that can be empirically tested.

Given this philosophical predicament, we require a naturalistic model of how some desires, beliefs and intentions arise without rational deliberation. But when we say “naturalistic,” we do not mean some kind of *illiberal* reductive or non-reductive materialist or physicalist explanation, whereby human intentional action is either nothing over and above the causally efficacious interplay of fundamentally physical factors, or else floats epiphenomenally above that causally efficacious interplay as a causally inert by-product of it, as it were a mere shadow-play of blind forces.

What is required, then, is a *liberal* naturalist account that is fully cognizant of:

i. The organicist/organismic basis of action-shapers, following EE and TTS,
ii. The weak yet salient influence of action-shapers and action-spaces on the formation of choices and intentional acts, which leads us to (iii):
iii. A fuller appreciation of the action-shaping web that grounds our spontaneous effective desires, our choices, and our intentional acts in our essential embodiment.

This philosophical picture is a full-out rejection of two assumptions that underlie TDM. First, it rejects the idea that choices and intentional actions as such can be treated apart from our the biochemical processes that unfold due to essential embodiment—that is, from the necessary and complete embodiment of our rational, self-conscious, conscious human minds in and throughout our living organismic animal bodies. The upshot of this is that choices and intentional actions cannot be deductively explained on the basis of motives or reasons alone; and neither is it possible to ascribe them to or primal will or primal volition.

All too frequently, the level of self-conscious deliberative motive or reason is where the explanation stops. Thus, if in a court of law, it’s established “beyond a reasonable doubt” that X murdered Y because X chose and acted out of intense jealousy, then this is a purportedly adequate and complete explanation that appeals to a self-conscious deliberative motive or reason.

But TDM does not explain how the psychological state “jealousy” arose. What were the physical factors that influenced its formation? In other words, as long as the causal and contextual factors in play are left unanalyzed, the explanation stops too early, and constrains itself to analyzing self-conscious motives and reasons only. This leads seamlessly into the idea that we can adequately and completely explain all choices and actions by appealing to some capacity for practical reasoning together with—or fueled by—self-conscious motives or reasons that collectively determine choice and intentional action.

But what would be the starting point of a liberal naturalist theory of action-shapers? We propose to start with the *neuro-immunological effective processes* that operate *inside and throughout* the living organismic body of the essentially embodied intentional agent.

## Neuro-Immunological Effective Processes

To see how the action-space is shaped, let’s start our account with introducing some evolutionary mechanisms that are active in our bodies. Empirical research has shown that the “behavioral immune system” evolved from mechanisms that facilitated behaviors that minimized infection risk and enhanced fitness (Schaller et al., 2015). For instance, disgust has been long recognized as a primary motive of defensive response to a threat posed by microscopic pathogens (Kupfer et al., 2021), while itch-generation mechanisms and grooming behaviors may have evolved to defend ourselves against ectoparasites (which attach to a host’s surface/skin) (Kupfer and Fessler, 2018). Hence, these findings indicate that our actions stem from evolutionary mechanisms that are not always explained by first-order conscious motives or rational deliberation. However, in the case of the defense against ectoparasites, we can already see that the immune system is involved in such mechanisms.

The nervous and immune systems have long been considered as compartments that perform separate and different functions (Aarli, 1983). However, recent clinical, epidemiological, and experimental data shows a wealth of evidence that the nervous system receives messages from the immune system and vice versa. As (Nutma et al., 2019) describe: many molecules associated with the immune system are widely expressed and functional in the nervous system and vice versa (Ziemssen and Kern, 2007). It has become evident that cross-talk along the gut-brain axis regulates inflammatory nociception, inflammatory responses, and immune homeostasis (Agirman et al., 2021). The complex interaction between nervous system and immune response contributes to e.g., neurodegenerative diseases, neuropsychiatric disorders, peripheral nervous system and neuro-oncological conditions, as well as aging, but also contributes to mechanisms of regeneration and repair (Nutma et al., 2019).

An immune response, with the inflammatory response as an example, may be localized or systemic, and even localized responses are often accompanied by systemic responses, coordinated by the nervous system, such as fever and white blood cell production (Besedovsky et al., 1983; Kennedy, 2010; Dhabhar et al., 2012). So, when someone cuts their finger, the local immune system responds directly and locally, but also sends out a hormonal or neuronal signal to the central nervous system which coordinates systemic support to the wounded area.

Given this evidence, it does not make sense to artificially separate the nervous system and the immune system into two distinct systems.

The major evolutionary function of the immune system has been to protect its host from hazardous environmental agents such as microbes or toxins, thereby preserving the integrity of the body (Schultz and Grieder, 1987; Kaufman, 2010). In executing this function, it has the ability to adjust metabolic rates (Odegaard and Chawla, 2013; Zmora et al., 2017; Alwarawrah et al., 2018), set off hormonal (chain) reactions, redirect energy from one organ system to another and to issue neurological responses to perceived threats (Chu et al., 2021; Salvador et al., 2021). Evolutionary development has selected for this ability to respond properly to these threats including stressors like predation or natural disaster, for instance by increasing the delivery of oxygen and glucose to the heart and the large skeletal muscles (Cannon, 1932).

For instance, when one needs to outrun a predator or deal with a dangerous animal like a snake, oxygen and energy will be redirected to the systems that need it the most at that particular moment (for instance, the heart, lungs, legs, or arms). At the same time, it directed away from functions that are at that moment not required.

To orchestrate the response to any threat, our nervous system and immune system closely work together, a feature that is visible in the brain. Three regions of the brain direct the stress response: first, the amygdala, which detects threat and triggers the fight-or-flight response; second, the prefrontal cortex, which helps us deal calmly with stress, and can prevent or shut down a freeze, fight-or-flight response; and third, the hippocampus, which supports stress recovery (McEwen and Gianaros, 2010).

As established by Dhabhar et al. (2012) and Dhabhar (2018), an acute or short-term stress response induces increased circulating concentrations of three principal stress hormones: (I) norepinephrine and (II) epinephrine in first instance, followed by (III) corticosterone. This circulation of hormones is accompanied by a rapid redistribution of immune cells among different body compartments (Dhabhar et al., 2012). Since any response (freeze, flight, or fight) may result in risk of injury and subsequent entry of infectious agents into the bodily perimeter, inflammatory responses in the immune system that accelerate wound repair and prevent infections have been evolutionary selected-for (Williams and Leaper, 1998). In normal, healthy, circumstances this inflammatory response is self-regulating and naturally resolves itself (Edwards and Guilliams, 2010; Dhabhar et al., 2012; Sugimoto et al., 2016; Balta et al., 2017; Dhabhar, 2018; Serhan and Levy, 2018). After encountering the snake or drinking from a stagnant waterpool, the body might set off an immune reaction, but in normal circumstances, this process is self-terminating.

Human physiological responses like the “flight or fight” still reflect the dangers and demands of earlier selection environments. Therefore, threats that do not require a physical response may still cause physical effects, including changes in the immune system (Segerstrom and Miller, 2004; Campbell and Ehlert, 2012). Hence, the embodied neuro-immune response system does not discriminate between immanent threats in the environment (such as encountering a snake) and stressors like having to sit an exam. Both events may thus be accompanied by the same physiological response, i.e., increased (nor)epinephrine and corticosterone levels in the blood, increased heart rate, breaking out in cold sweats, dilated pupils, mild tremors and a feeling of anxiety (Campbell and Ehlert, 2012).

Many of those physical effects have some advantage to deal with the potentially dangerous situation: the entire body goes as it were into “alarm mode” and is poised to act. The dangerous situation shapes the action-space, with certain courses of action being highlighted over others, as humans’ defense cascades are dependent on subjective representations of body states and the meaning they attribute to previous experiences (Kozlowska et al., 2015). In the case of encountering the snake the highlighted response could be quickly to evade the animal, to strike it with nearby object, or to freeze, depending on the person’s perception of the threat and their previous experience. In the case of taking the exam, the stress hormones most likely will increase focus, although the response-cascade physically drains the available energy in the body quite quickly, as resources such as oxygen or glucose are redirected to the brain (Campbell and Ehlert, 2012).

The degree of anxiety that individuals experience in a stressful situation differs. Research has shown that individuals with low levels of anxiety experience increased risk avoidance when primed with emotional cues, compared to highly anxious individuals (Charpentier et al., 2016). Even when both groups encountered similar threatening conditions, their choices significantly differed. High levels of anxiety correlates to higher neuronal activity in the amygdala (Davis et al., 1994; Sehlmeyer et al., 2011) which influences both the flexibility to act and impulse control, which may lead to exaggerated responses and the inability to adjust one’s behavioral course (Moustafa et al., 2017). Conversely, chronic exposure to low levels of stress may lead to a habituation of (mild) risk-avoiding behavior (Charpentier et al., 2016; Matisz et al., 2021).

So, it is clear that in a given situation, people choose different options. Even when an agent might perform various actions equally well in a given situation, only some actions are highlighted to be so desirable from a first-person standpoint that they appear as the only feasible possibilities. In other words: within the action space, certain actions are highlighted as desirable or preferable, as opposed to other alternatives. As we’ve indicated, the capacity for choosing to perform an action is not removed, but diffusely and saliently influenced.

As explained by Rodrigues et al. (2004), stressful experiences and associated changes in the release of stress hormones produce both useful and counterproductive effects on the hippocampus, hypothalamus, amygdala and other brain regions throughout life. Memories formed during emotional experiences are stored for future use in similar situations. For example, if we are injured, we acquire information about the stimuli that were associated with the event so that we may avoid harm later on (Rodrigues et al., 2004). However, as these authors describe, dealing with stressful situations does not necessarily lead to adaptation, and beneficial forms of learning that promote future resiliency. Stressful experiences can just as well lead to changes in physiological, neural and cognitive processes, conditioning an individual to respond in certain ways to a given situation. In turn, these effective processes change behavior. In some cases, it is the presence of such processes that makes one vulnerable to stress-related complaints (McEwen and Gianaros, 2010).

For instance, and staying with the example of conditioning, suppose someone has traumatically encountered a snake in an otherwise peaceful meadow. However, every time this person crosses a meadow-like environment, her alarm system is already primed, and even innocuous events like a movement in the grass might trigger stress responses.

Again, here is the brain involved: the amygdala is responsible for learning behavior, but it is also involved in regulating subjectively experienced feelings of fear and anxiety (Davis and Whalen, 2001; LeDoux, 2003; Costafreda et al., 2008; McEwen and Gianaros, 2010). In other words: an otherwise peaceful meadow now appears as representing a hazard or potential danger after the snake-incident. Evolutionary speaking, it is clear why this connection is established.

However, if this process misfires, persons may become too primed and start to perceive danger everywhere. So, a given perceptual state might trigger all kinds of modifications in the body. As past experiences shape stress responses, it follows that they (A) actively thought-shape our perception via the cognitive attitude of the imagination (i.e., resulting in beliefs about the world) and (B) actively action-shape our options (i.e., resulting in intentions and desires for acting in the world) through the conative attitude of the imagination, combined with affects and emotive cues.

It is important to repeat here that our body’s stress response is the same for “real” threats such as pathogens or predators as for “perceived threats” (e.g., worrying about sitting the exam, how to pay the mortgage, suspecting a snake to lurk in the grass, or believing someone is out to get you).

When we continuously worry, and negative thoughts continuously occur in our mind, or if we find ourselves over prolonged periods of time in a dangerous situation (e.g., war), we are exposed to chronic stress, defined as an extended period of exposure to potentially threatening or emotionally challenging stimuli that exceed our allostatic load (Sterling et al., 1988).

When we are exposed to chronic stress, changes in behavior and morphology of the amygdala occur (Boyle, 2013). Chronic and/or repeated stress causes remodeling of brain circuitry (McEwen and Gianaros, 2010; Zhang et al., 2019), which may even lead to specific synaptic plasticity within the amygdala. In turn, this change in plasticity may lead to counterproductive responses that can even result in psychiatric syndromes such as anxiety and depressive disorders (Shekhar et al., 2005). The acute response to stress, embodied in the “fight or flight reaction,” induces a temporal state of anxiety. But when stress is prolonged, depression (amongst others) may develop insidiously and gradually under the cloak of continuing anxiety symptoms (Wheatley, 1997; Chekroud, 2015). We provide a hypothetical example of how this process might play out.

First, imagine someone who habitually experiences chronic stress at work, afraid of losing his job, and with that, not being able to pay his bills. He may become trapped within a chronic, self-reinforcing stress loop: his fear to lose his job makes him experience his work environment as dangerous or hostile, hence reinforcing specific (neurologic) processes within his brain. In turn, these very processes cause him to be even more alert and afraid. As a result, he slowly slips into a state of depression (Caspi et al., 2003; McEwen, 2003). Due to his depressed frame of mind, he may be drawn to foods that encourage serotonin production, like sugar-rich and refined carbohydrate foodstuffs—so-called “comfort food” (Inam et al., 2016). Serotonin plays an important role in regulating one’s mood (Young and Leyton, 2002; Jenkins et al., 2016), even to such a degree that an imbalance of serotonin may contribute to depression (Carr and Lucki, 2010; Jones et al., 2020). Hence, such food cravings may be an immunological attempt to restore the bodily homeostatic balance.

And though this so-called “comfort food” may elevate one’s mood for a short while, if the underlying causes of the depression are not removed [for instance, tryptophan shortage (Cowen and Browning, 2015) or thyroid disfunction (Bauer et al., 2008)], this person may get stuck in a vicious circle of food craving, resulting ultimately in altered neural plasticity (Jabeen and Haleem, 2008; Benton, 2010; Beecher et al., 2021). Even when self-consciously knowing that a walk would release mood-enhancing endorphins and other natural brain chemicals (for instance, endogenous cannabinoids) which would enhance a sense of wellbeing, thereby delaying cognitive degradation (Zhao et al., 2020), deciding to actually start exercising or adhering to a training regimen may be extremely difficult. It could be that the person trapped in a chronic stress loop cannot effectively align or configure his bodily systems in such a way that he actually puts on his shoes and goes outside.

Likewise, while this person could in theory refrain from eating sugar-rich and refined carbohydrate food, and instead could get up from the couch and take a walk, in practice, this could prove difficult due to the active processes in the body (Molteni et al., 2002). Again, the cascade of neuro-immunological processes prevents the body from aligning its mental and physical systems in such a way that a given course appears as promising or even remotely attractive.

It is not that a person in this state of mind and body is physically incapacitated to the point of their not being able to perform a given action, but instead that the capacity to translate intention into action (from thinking: “it would be nice to have a walk” to actually going outside and walking) is heavily impaired.

What happens in this example is that two cascades of neuro-immunological causes and effects dynamically influence this person’s mood, and consequently shapes his action-space.

In normal circumstances, hormonal cascades are inhibited via a negative feedback loop. In other words, such processes naturally self-terminate (Edwards and Guilliams, 2010; Dhabhar et al., 2012; Balta et al., 2017). However, due to the fact that our bodies have evolved in an environment with a different evolutionary fit than they inhabit now, neuro-immunological processes that were intended to self-terminate may be inadvertently trapped in a self-sustaining loop (Zilberter and Zilberter, 2017; Freeman et al., 2018; Pruimboom and Muskiet, 2018).

So, the immune system has a direct and salient, even if relatively diffuse effect on the body and on the scope of the action space. Note also that these processes interact with thought-shapers through the imagination (culminating in beliefs or imaginings) and action-shapers (culminating in intentions and desires or lack thereof).

Put concisely, and combining the claims we’ve introduced earlier in this section, we can construct the following speculative picture: a person in which neuroplasticity, learning capabilities, and cognitive processing are impacted by high levels of sugar intake will experience also increased stress levels, which in turn results in even more impact on neuroplasticity, learning, and cognition. So, it seems plausible that if the bodily stress system is continuously exposed to a diet with high sugar contents, the first cascade (anxiety, chronic stress) is enhanced by the second cascade (diet, altered neuroplasticity, resulting in more anxiety).

An additional influence is that the resulting behavioral patterns may easily induce the nocebo effect (i.e., negative expectations lead to outcomes that are worse than necessary) (Barsky et al., 2002; Brazil, 2018; Colloca and Barsky, 2020). Evidence suggests that stress induces heightened sensitivity to pain and vice versa (Benedetti et al., 2006, 2020). But equally, the nocebo effect plays a role in perceiving the environment, and conceptualizing the future (Colloca and Barsky, 2020). Put differently, the nocebo effect exert itself also via thought-shapers and action-shapers.

Summarizing, the combined effects of the two cascades and the nocebo effects have been be synoptically connected with the help of evidence cited in the previous paragraphs. If events in the environment are represented as dangerous, and if one is simultaneously in a depressed our anxious state of mind (Wells and Kaptchuk, 2012), the action-space is inflected in ways that show how the capacity to take action, or to select one action over another is severely affected or limited.

Neuro-immunological processes function also in the reverse direction. Or, to put in the language of TTS, when subjected to generative thought-shapers, the action-space can expand, and possibly even expand beyond what one at any given point could imagine. Through the concept of mental rehearsal (repeatedly imagining performing an action, and also imbuing this imagined activity with positive affect), the circuits in our brains can reorganize themselves to reflect our very intentions (Dispenza, 2012, p. 73).

All this does not mean that we’re advocating any kind of biological determinism, where individuals are inevitably victims of blind biochemical, endocrine and humoral processes that play out in their bodies. To think so would amount to import the categories and thought-shapers of the TDM picture in through the backdoor. In fact, the interplay of psychological and physical influences on the action space unfolds non-linearly and non-deterministically. So, while we can identify certain effective processes and retroactively construct a chain of causes and effects, many or even most of these effects are best understood as only exerting degrees of influence, not as exerting strict, entirely computable, one-directional determination.

Equally, to frame our theory of action-shapers as a form of biological determinism would be seriously to misinterpret the picture we are sketching out here, and would ignore the recent strong neuroscientific evidence to the effect that we can gradually affect the structure of our brains by thinking and feeling differently.

In other work, we have discussed the meta-cognitive shift that literally causes people to perceive, act, and think radically differently as *creative piety* (Hanna and Paans, 2022, in press). That is, the capacity to “step outside” the mental confines in which one seems stuck and adopting a transformatively different higher-dimensional or higher-order perspective. To practice creative piety means to adopt a different set of mental habits to circumvent the inhibiting influence of mechanistic, constrictive thought-shapers and action-shapers alike.

It should be emphasized that the influence of neuro-immunological effective processes is –in many cases– positive. For instance, as members of certain religious traditions (e.g., Buddhism), military traditions (e.g., special forces) and sports-training traditions (e.g., long distance running) have long known the positive effects of meditation (Hoge et al., 2018; Tolahunase et al., 2018), intermittent fasting and short term exposure to cold or hot temperatures (Mattson, 2014). By themselves and combined they have shown to improve cognitive functions and resilience to stress, decrease inflammatory markers in the blood, and aid metabolic flexibility, cell growth and hence muscle regeneration (Mattson, 2015; Freese et al., 2017; Mattson et al., 2018; Pruimboom and Muskiet, 2018). All these factors add up in feeling energetic and being ready to act. But equally, they lead to the exact reverse effect of the constrictive loop we have been exploring in the preceding paragraphs (Pruimboom and Muskiet, 2018).

## Discussion: A Model of Action-Shaping

As outlined in the previous section, a variety of occurrent neuro-immunological effective processes shape, orient, and/or narrow the action space in real-time. Therefore, we can approach them as effective causal influences that result in or contribute to the formation of thought-shapers and/or action-shapers. That is, they are nonlinear, non-deterministically causal, but fully embodied influences on the actual and/or perceived range and/or type of actions that persons subjectively experience as having at their disposal.

Summarizing the preceding discussion, as forms of self-presentation engendered by the body, action-shapers influence one’s perception of the available actions at one’s disposal, although this representation is by no means always veridical. As also indicated previously, action-shapers are felt and experienced rather than consciously or deliberately imagined or believed. This makes them extremely powerful and effective, as they shape not only the capacity to *want-to-perform* certain intentional actions, but they also influence the neuroplasticity of the brain. Or, as Donald Hebb memorably said: “Neurons that wire together, fire together.” And so, habitually performing an action or abstaining from doing so reconfigures the brain structure. Literally, we shape our mind by shaping our brain if we act or abstain to act in certain ways. Consequently, it can become harder to break certain habits or to make a certain choice. Conversely, it can also become easier to perform certain acts if the mind is habituated to do so.

If we imagine the action-space as an abstract space of possibilities, we can see that thought-shapers and neuro-immunological processes function as forces that push and pull the shape of the action-space. It is important to keep in mind that this process unfolds organically and dynamically and over time. The upshot is that the scope and size of the action-space varies over time, according to our allostatic load, and throughout various hormonal cycles of the body, that in turn change over time.

This model of looking at action-shapers and the action-space has theoretical implications for both TDM and another theory of action, namely Brian O’Shaughnessy’s (O’Shaughnessy, 2008, p. 511) account of “embodied trying” as the formation of a will-act. If we “try” to do something in this qualified sense, we orient various systems (cognitive, motor, and hormonal) in our bodies toward performing that particular action or achieving that particular goal (Hanna, 2021, p. 319).

However, if immunological effective processes are action-shaping in the very earliest stages of aligning and configuring these various bodily systems, then the very attempt to “try” is either (I) already stopped, stunted, or redirected, or (II) enabled, enhanced/intensely focused from the very beginning.

As explained in the third section, TDM has trouble explaining why certain desires arise if it cannot fall back on either (a) deductively explain the deliberation process leading up to a certain action, or (b) some kind of primal will or volition that is the source of all intentions and desires. The prime drawback of TDM seems that it relies on a deductive model that attempts to identify a primary source for actions. Of course, even while using this method, one could explain the inappropriate behavior of Smith at the bar last night by citing multiple causal influences (“Mr. Smith had a stressful day on his job, *and* hit the curbstone with his car while parking, so he was already angry when he entered the bar….”). But even so, TDM utilizes a deductive method that has to fully explain Smith’s actions. It searches for the root cause of Smith’s actions by examining a series of factors that can be included in a deductive account.

The model of O’Shaughnessy recognizes the complexity of forming a “will-act,” and casts the act of “trying” and effectuating the intentional action as two synchronous events, or as an “ontologically fusion” of sorts:

“It is trying that efficaciously causes the intentional body movement, but not as such or *per se*, rather only as ontologically fused with a larger causally efficacious dynamic process, because the conscious event of trying constitutes only a proper structural part of the whole dynamic process. So the metaphysical picture is one of individually necessary and individually insufficient but jointly sufficient synchronous and ontologically fused event-causation” (Hanna, 2021, p. 320).

This idea somewhat resembles the philosophical concept of an “assemblage” (Deleuze and Guattari, 2011, pp. 7–8, 337, 375). An assemblage is a composite entity that consists of various parts or agents (or both) that interact in such a way that they jointly function for all intents and purposes as a single, unified entity. However, in all cases, the total is more than the sum of the parts, and so the assemblage can accomplish feats that any of its individual parts cannot. Examples are the fusion between a car and a driver, or a horse and a rider, or a computer and a human being.

We can usefully combine the concepts of assemblage and ontological fusion to elucidate the action-shapers model. Consider a musher who drives a dog sled and directs the sled dogs to follow a certain trail. However (and from personal experience) we know that sled dogs can be notoriously stubborn. So, the musher forms in the first instance a new unity (an ontologically fused “assemblage”) with his dog team and the sled. He directs the dogs, exerting various causally efficacious influences on them. However, the dogs do the actual pulling and provide the physical force to move the sled over the ice. At some point, the driver directs the dogs to the left, but—stubborn as they are—the entire dog team decides to take a right turn. The musher still exerts his influence on the dogs by pulling, shouting instructions or leaning to the left. However, the dogs exercise their own agency, which is (I) shaped by the weight of the sled, the leaning of the driver and the fact that they are attached to it, but (II), as the dogs realize all too well, they can exercise effects over and above these constraints.

In much the same way can we conceptualize the relation between action-space and act. If one would like to perform some act C, it can still be made very difficult by the dynamically contouring action-shapers that constrain, widen or orient one’s action space, even to the point where performing act C appears as being physically impossible. However—and this is where O’Shaughnessy’s model leaves us in the dark—there can be all kinds of obstacles and accelerators in the relation between “trying” and intentional movement or lack thereof. The assemblage—like the dog sled—does not always function smoothly. Moreover, due to being an organic assemblage, the relation between body and action is not of the same type as that of a driver to a car, but is more akin to the relation between a musher and his sled dogs. Our body, as we explained in the preceding section—is an organic entity in which multiple processes simultaneously play out in real time, some of which we may not consciously control or direct, and some of which occur as the result of evolutionary misfiring. And, in the case of the self-sustaining loop, a neuro-immunological process might “behave” like a stubborn husky, seemingly refusing to adapt to a new situation.

We can still identify another type of influence in the action space. Consider the musher again, leaning to the left and trying to convince his dog team to follow his lead. After considerable insistence, some dogs finally obey the instructions, and suddenly start pulling to the left. However, due to the quick change of direction this causes, the sled slides sideways on the slippery ice, describes a semi-circle and comes to a sudden halt against a nearby tree. What happens is that the imbalance set off by the stubborn dogs and the consequent response of the musher takes place in an environment with certain features—in this case, the presence of slippery ice underneath the sled.

This slipperiness makes a sled pulled by sled dogs an effective means of transportation in Arctic environments, but it also contains a latent danger: given the right circumstances (i.e., sudden shifts and stops) the very feature that causes mushing to be so efficacious becomes a potential danger. By analogy, and as we explained, some neuro-immunological responses likewise become dangerous and exert long-term effects that one might overlook altogether when thinking about intentional actions. Their organic function is fully explainable for some, evolutionary selected circumstances. However, given certain environmental pressures (such a chronic stress or exposure to a man-made substance like asbestos), such processes may misfire. Yet, they exert diffuse yet salient effects. Often, such effects are overlooked because they gain momentum over long time scales. To see why, we have to leave the example of the sled dogs and return to Smith’s behavior at the local bar.

In that example, we showed how TDM searches for the cause or causes of Smith’s behavior by deductively constructing causal explanatory chains. However, such explanations remain often stuck on the level of observable events, such as Smith having a stressful day at work, or him hitting the curbstone while parking his car. By contrast, the theory of action-shapers can (I) easily concede that such factors *could* have played a role in Smith’s inappropriate behavior, but (II) it can also *extend* the explanatory account to include factors that pertain to Smith’s embodiment.

For instance, consider the fact that Smith has not liked his job for years, and this may have caused a chronic stress build-up, and a reduced allostatic capacity, triggering and physically altering his amygdala. In response, Smith started to crave sugar-rich comfort food. But slowly, over the course of a few years, Smith has been developing symptoms of clinical depression. In turn, this depression has severely reduced his feeling of self-worth, a situation that is not really helped by him gaining weight due to his dietary choices. The bodily environment of Smith has primed him already to adopt certain actions over others. The sugar in his food has made him increasingly grumpy and moody, and even more prone to stick to “old habits.” Where Smith might have been an aimable person in usual circumstances, the combined collision with the curbstone and his stressful day at work negatively reinforce his already low self-image, his chronic stress and trigger his tendency to moodiness (Dantzer et al., 2008). Events that would have gone by unnoticed in normal circumstances, trigger the “organic assemblage” Smith in exactly the wrong way that fateful night at the bar. What happens is an ontological fusion between multiple neuro-immunological, mental and physical factors that jointly determine Smith’s action space.

With the numerous examples and the notion of action-shapers we discussed, we can return to our opening statement: not of all intentions translate into actions. But now, we can understand why some intentions remain just that. In particular, our essential embodiment provides action-shapers that determine our action space. In turn, action-shapers—in conjunction with thought-shapers—are the dynamic forces that enable or impede our capacity for acting. While some action-shapers are generative and support the capacity to act, others are constrictive and diminish it. With this theory, we have developed a rudimentary model for thinking about free will and choice that does not just depart from motives and reasons, but equally from neuro-immunological processes that occur in our bodies. Put differently, the theory of action-shapers provides an alternative model for thinking about agency that departs from an organicist foundation and the essential embodiment of human agents, instead of the linear casual chains characteristic of TDM.

Of course, all kinds of additional questions about moral responsibility and the principle of alternate possibilities can be raised. However, the main point of our theory is that instead of searching for a prime motive or root cause, we might do well to adjust and broaden our perspective and regard the action space as the organic, embodied and dynamically contoured environment in which action-shapers highlight certain courses of action over others. In conjunction with thought-shapers and *nocebos*, this mix dynamically and non-linearly determines our behavioral patterns and habits, up to and including changes our brain structure and the capabilities for acting we have at our disposal.

If the foundations of the theory we have provided here are correct, we require further research in the precise dynamics of neuro-immunological processes and their influence on mental states and psychological experiences. Conversely, we might also require research in the ways how action-shapers can be used to aid or accelerate recovery processes, coping with grief or loss or overcoming potentially negative or inhibiting situations like depression, low self-worth, prolonged unemployment, or feelings of exclusion.

Concluding, we have attempted to provide a synoptic, fully naturalist picture of intentional action in this article. It is fully naturalist in the sense that it takes the findings of cognitive science and immunology fully seriously. However, it is not reductionist, physicalist, mechanistic or illiberal in the sense that any form of biological determinism is endorsed. And neither is it dualist in the sense that it drives a wedge between mind and body. Instead, it is fully within the purview of both TTS and EE.

Indeed, there are many further questions that could be explored regarding this model, especially regarding issues surrounding moral responsibility, freedom of the will, the link between bodily and mental processes, the nature of causation and the role of self-presentation, and the relation between empirical findings and speculative theorizing. However, we hope this article contributes to an organicist understanding of our capability to act.

## Author Contributions

OP developed the overall line of argument and philosophical materials and TTS. BE provided numerous references and ideas and concepts with regard to immunology and their relation to the overall argument. OP and BE wrote the section on neuro-immunological processes and the conclusion in close collaboration, with equally sharing the workload and writing and editing tasks. Both authors contributed to the article and approved the submitted version.

## Conflict of Interest

The authors declare that the research was conducted in the absence of any commercial or financial relationships that could be construed as a potential conflict of interest.

## Publisher’s Note

All claims expressed in this article are solely those of the authors and do not necessarily represent those of their affiliated organizations, or those of the publisher, the editors and the reviewers. Any product that may be evaluated in this article, or claim that may be made by its manufacturer, is not guaranteed or endorsed by the publisher.
